# Supplementary material for: Cyto-nuclear shuttling of afadin is required for rapid estradiol-mediated modifications of histone H3
Source: Neuropharmacology. 2018 Dec;143:153–62. doi: 10.1016/j.neuropharm.2018.09.041 (PMC6277849; doi:10.1016/j.neuropharm.2018.09.041)
Supplement: Sellers_et al._supp-information_R2.docx [file mmc1.docx]

**Cyto-nuclear shuttling of afadin is required for rapid estradiol-mediated modifications of histone H3**

Katherine J. Sellers^1,2^*, Iain A. Watson^1,2^*, Rahel E. Gresz^1,2^, Pooja Raval^1,2^, Deepak P. Srivastava^1,2^§

**Supplementary Information.**

**Supplementary Figure 1. Validation of afadin antibody specificity. (A)** Representative confocal images of DIV 26 cortical neurons transfected with a control shRNAi construct, or an shRNAi specific for afadin (Srivastava et al. 2012). Following 7 days of transfection, cells were fixed and immunostained for GFP (morphological marker) and l/s-afadin (Sigma, cat. No. A0224). Yellow dotted lines outline soma of GFP-positive cells; thus Ctrl/afadin-shRNAi positive; blue dotted lines outline soma of GFP-negative cells. **(B)** Assessment of afadin expression in soma of GFP-positive cells demonstrated a significant decrease in afadin staining in presence of afadin-shRNAi compared to ctrl-shRNAi, and GFP-negative cells (data not shown). (***, p < 0.001, Student t-test; n = 9-11 cell per condition from 3 independent cultures).

**Supplementary Figure 2. Full length scans of western blots. (A)** Full length scan of western blot from Figure 1 C; red dotted box indicates portion of western shown in main figure. **(B)** Full length scan of western blot from Figure 1 E; red dotted box indicates portion of western shown in main figure.

**Supplementary Figure 3. Full length scans of western blots. (A)** Full length scan of western blot from Figure 2 C; red dotted box indicates portion of western shown in main figure. **(B)** Full length scan of western blot from Figure 5 C; red dotted box indicates portion of western shown in main figure.
